# Flow phenomena in laminar flow through streamlined and sharp-edged short monolithic structures

**DOI:** 10.1038/s41598-023-42568-2

**Published:** 2023-09-21

**Authors:** Mateusz Korpyś, Marzena Iwaniszyn, Katarzyna Sindera, Mikołaj Suwak, Anna Gancarczyk, Andrzej Kołodziej

**Affiliations:** 1 Institute of Chemical Engineering, Polish Academy of Sciences, Bałtycka 5, 44-100 Gliwice, Poland; 2 Faculty of Civil Engineering and Architecture, Opole University of Technology, Katowicka 48, 45-061 Opole, Poland

**Keywords:** Engineering, Chemical engineering

## Abstract

Monolithic structures of catalytic reactors offer low flow resistance, but their drawback is weak heat and mass transport. For transport intensification, innovative streamlined structures were designed, the walls of which are shaped like an airplane wing. Extensive CFD (Computer Fluid Dynamics) studies were performed for the streamlined and—for comparison—classic (sharp-edged) structures, covered flow phenomena, and heat transfer to channel walls. The streamlined structures were made using the SLM (Selective Laser Melting) method to perform heat transfer experiments that gave a satisfactory agreement with the CFD. Heat transfer for streamlined structures was, by CFD, more intensive than for the classical ones. CFD simulations showed a significant reduction of vortices in streamlined structures. The lack of an inlet vortex was demonstrated, for classic structures strongly limiting transfer properties. For the streamlined structures the outlet vortex even intensifies heat transport near the outlet of the channel. The CFD showed the flow patterns for the structures as well as the distribution of transport coefficients within the millimetre-sized channels.

## Introduction

In recent decades, catalytic monolithic reactors became the standard and were widely applied as automotive exhaust afterburners, with other industrial and environmental applications. The main advantages of monoliths are large specific surface and low flow resistance. The first monoliths were made of ceramics (cordierite), and soon other materials like graphite, alumina and metals appeared^1,2^. The monolith consists of many straight, parallel capillary channels; a catalyst is deposited on the channel inner walls. In a few millimetres wide channels (4 mm down to less than 1 mm), several dozen centimetres long, mainly developed laminar flow takes place^3^. In the developed laminar flow, the streamlines (velocity vectors) are parallel throughout the channel, and the heat (mass) transport takes place by conduction (diffusion).

The advantage of the developed laminar flow regime is low flow resistance: the product of the Fanning friction factor and Reynolds number, $(f\cdot \mathrm{Re})$, is constant^4^. However, the heat (mass) transport intensity is rather low; the dimensionless Sherwood and Nusselt numbers are constant and do not depend on the Reynolds number (flow velocity). This may result in reduced process yield, especially for fast reactions, e.g. catalytic afterburning at higher temperatures, due to insufficient mass transport of the reactants to the catalyst surface^5^.

The process efficiency in the catalytic reactor cannot be greater than the mass transport to the catalyst—only those molecules may react which have reached the catalyst surface. The heat and mass transport in reactors are closely related on the one hand by the Chilton-Colburn analogy^6^ and by the reaction enthalpy, on the other. Transport intensity is therefore significant and difficult to overcome limitation of the reactor yield. Whereas, the problem of flow resistance can usually be overcome by adding appropriate compressors, although the energy costs for pumping the fluid must be taken into account here.

However, in the inlet zone of the capillary channels, a so-called developing laminar flow occurs, which is characterized by much higher values of Sherwood and Nusselt numbers, depending on the Reynolds number^4^. The division of a long capillary channels into a series of shorter elements, in which mainly the developing flow takes place, makes it possible to achieve much more intense mass and heat transport, and thus—in many cases—higher process efficiency. Such structures have been called short-channel structures^5^. The flow resistance is a bit higher in such structures, but the gains from intensifying heat and mass transport are greater. While in a fully developed laminar flow increase of the transport intensity is in principle basically impossible, the short channel structures open the possibility to increase the transport by an order of magnitude thus avoiding the process yield limitation^7^.

**Figure 1:**
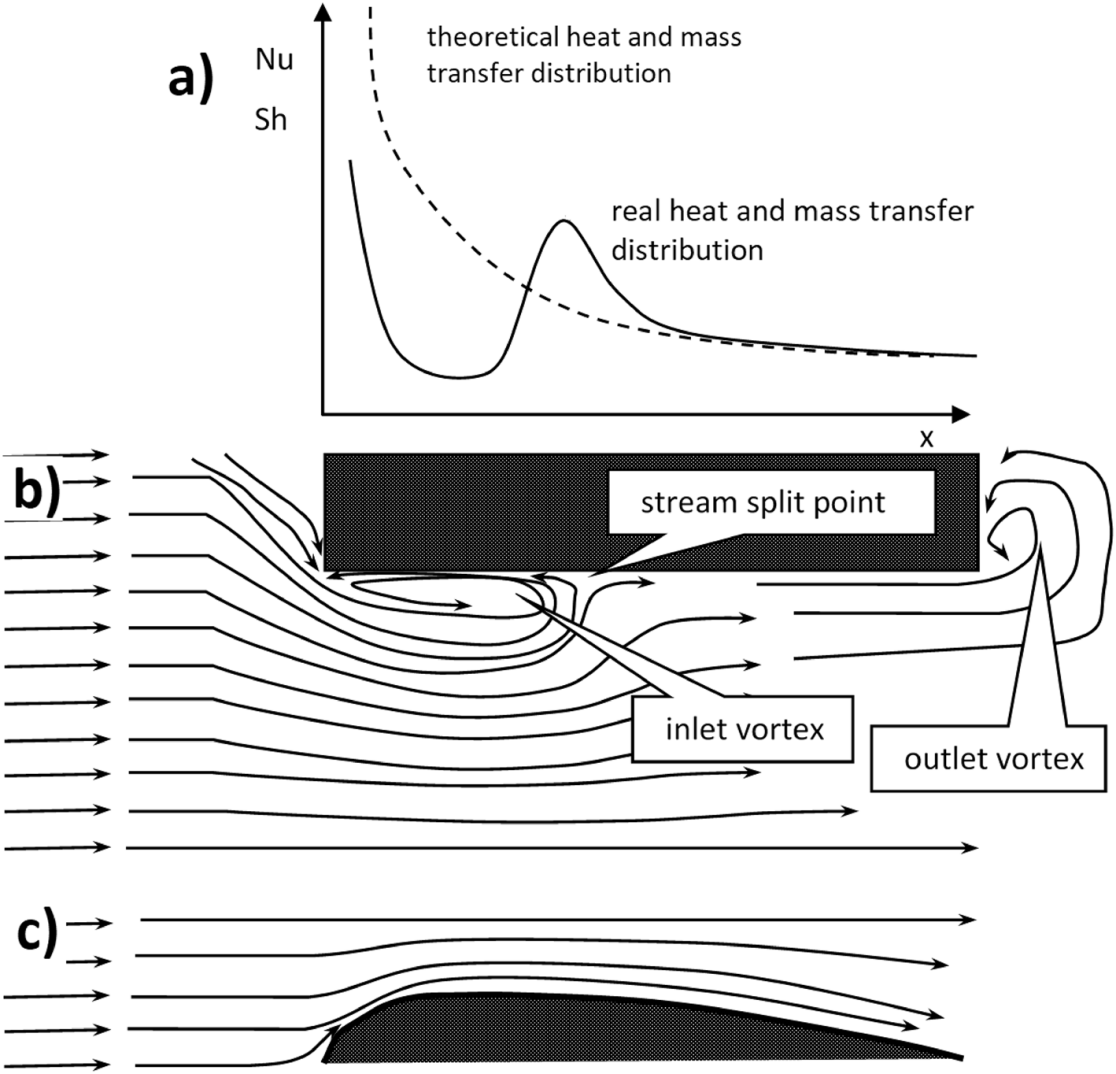
Laminar flow phenomena and transport coefficients for the structures studied: (**a**) transport intensity along the channel; (**b**) streamlines and vortex formation for the classic sharp-edged structure (inlet vortex formation); (**c**) streamlines for the streamlined structure (no inlet vortex).

The development of laminar flow in the inlet zone of the channel is based on the formation of velocity, temperature and concentration profiles. Assuming flat profiles at the channel inlet, they gradually transform into the parabolic ones, typical for the developed laminar flow^4^. However, such a situation assumes the absence of transverse fluid velocity vector components that are perpendicular to the channel axis. This never happens because the channel walls—even very thin ones—nevertheless have a finite thickness, resulting in the appearance of transverse components of the velocity vector near the channel inlet. As a result, the velocity vector is inclined from the channel axis, which leads to the so-called inlet vortex (Fig. 1b). For some distance, the streamlines near the wall are opposite to the principal direction, between the channel inlet and the so-called stream split point. The vortex is located in a place where theoretically (assuming no transverse velocity vector components) the zone of the most intense mass and heat transport exists. This vortex somehow shields the channel wall, hereby the minimum appears in the distribution of the local Nusselt (Sherwood) number (Fig. 1a). This disadvantageous phenomenon causes a certain reduction of the transport intensity in the inlet zone, where—theoretically—the transport should be the most intensive. The problem is rather scarcely discussed in the literature, cf. e.g.^8,9^. Figure 1 shows the fundamental difference between the—formerly discussed—model laminar flow and the reality.

The only remedy for the formation of such inlet vortices seems to be the use of the so-called airfoil profile, i.e. forming the channel wall of the structure in the shape of an airplane wing profile. In such a situation, the velocity vector tends to be tangent to the “rounded” surface of the airfoil leading edge thus producing no inlet vortex (Fig. 1c). The distribution of the local transport coefficient is close to the theoretical relationship and the overall transport becomes more intensive (Fig. 1a). This article describes the study of square streamlined structure using Computer Fluid Dynamics (CFD) and experiments. While the experiments reflect the macroscopic functioning of the examined structures, CFD enables numerical simulation of flow and transport phenomena in the microscale, which is the main goal of the presented research. At the same time, the results of experiments are used to confirm the correctness of CFD calculations.

### Design and manufacture of the streamlined structure

**Figure 2:**
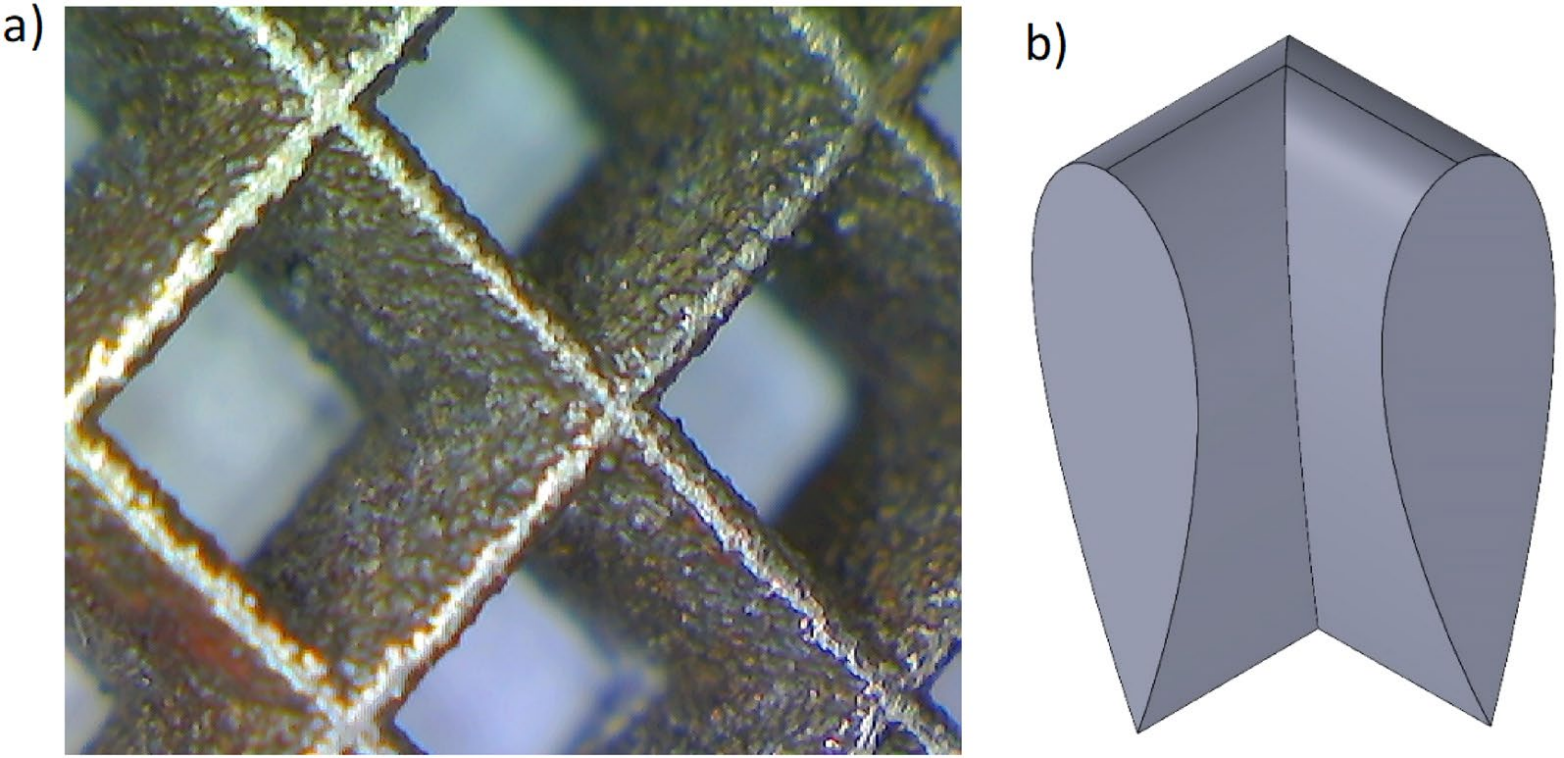
Picture (**a**) and the design sketch of the streamlined structure studied (**b**).

It was assumed that the streamlined structures would be a development of the short-channel ones, maintaining their ability to regulate heat and mass transport coefficients. The main change was the shape of the channel wall: its longitudinal section was similar to the airfoil profile (Fig. 2). It was expected that the streamlined structures would enable the elimination of unfavourable inlet vortices, thus improving the efficiency of heat and mass transport.

A channel with a square cross-section was studied. The single channel was 3 mm long with a side of 4 mm between the axes of the opposite walls. A dozen such channels have been combined into one rectangular structure measuring 45 mm by 30 mm and 3 mm thick (equal to the channel length). The specific surface area was ${S}_{v}$ = 925 m^2^ m^−3^, the void fraction $\varepsilon$= 0.71, and the hydraulic diameter of the channel ${D}_{h}$ = 3.1 mm. The streamlined shape of the wall is based on the airfoil profile NACA 0006. Extensive simulations of fluid flow and heat transfer phenomena were performed for the designed geometry of the streamlined structure using CFD software.

The streamlined structure design was implemented into a CAD program and then manufactured of powdered metal (stainless steel) using additive technology—selective laser melting (SLM). The geometry of the structure has been studied by X-ray computing tomography (CT) and compared with the CAD design. The difference between the design and the manufactured structure (according to the CT results) is less than 1% for ${S}_{v}$ and 4% for $\varepsilon$. The picture and design of the manufactured structure are shown in Fig. 2.

Additionally, to compare the phenomena of flow and transfer in the proposed streamlined structure, the classic short-channel structure with sharp edges has been designed with a geometry close to the streamlined one assuming the same void fraction $\varepsilon$ for both the structures. The assumed dimensions were: channel length $L$ = 3 mm, square channel width (between the wall axes) 4 mm, void fraction $\varepsilon$ = 0.71, specific surface area ${S}_{v}$ = 1036 m^2^ m^−3^ and the hydraulic diameter of the channel ${D}_{h}$ = 2.7 mm. The thickness of the channel wall was 0.63 mm, constant for the whole channel length. For the sharp-edged structure, only the CFD simulations were performed.

## Results and discussion

**Figure 3:**
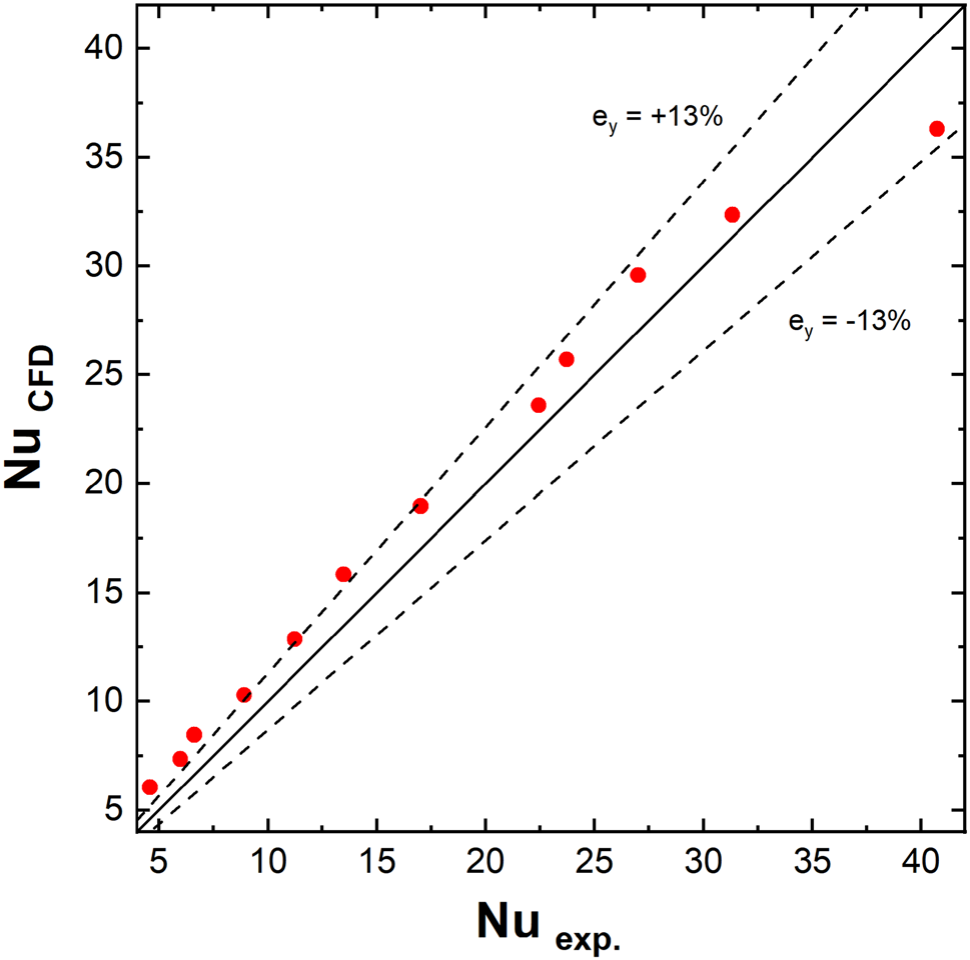
Comparison of Nusselt numbers for the streamlined structure obtained experimentally with those obtained from CFD calculations.

To verify the correctness of the CFD simulation, the results of the calculations were compared with the experiments (Fig. 3). The local values of Nusselt numbers calculated in the CFD program were averaged over the entire surface of the structure. Satisfactory agreement between CFD calculations and experimental results was obtained (average relative error, e_y_ =  ± 13%). It can therefore be assumed that the CFD procedure was carried out correctly.

The low differences between CFD and experiments may result from unavoidable experimental uncertainties as well as from the manufacturing method descripted in the above paragraph (cf. Fig. 2).

### Streamlined structure

**Figure 4:**
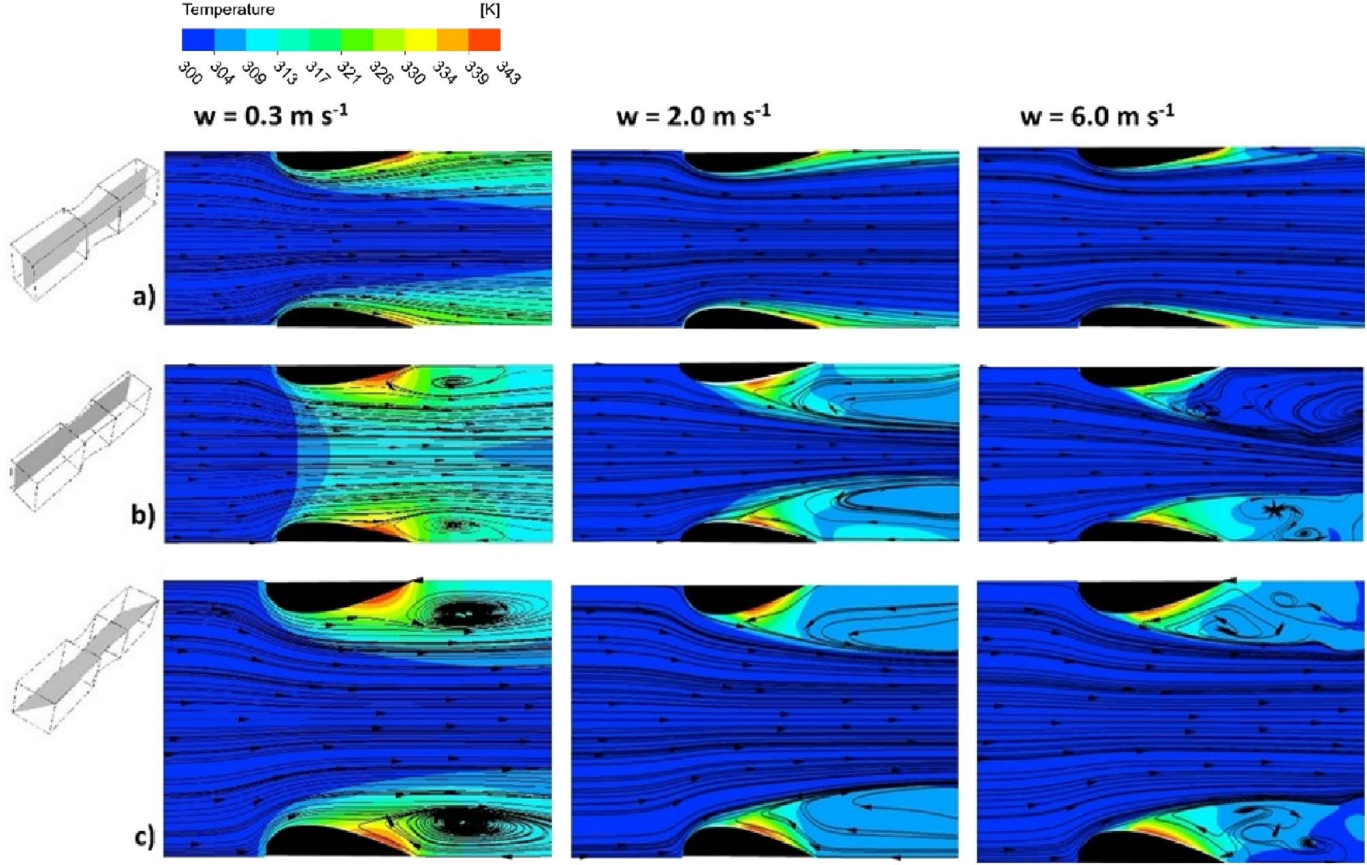
Streamlines and fluid temperature maps for the streamlined structure derived using CFD: (**a**) along the channel axis; (**b**) near to the channel wall; (**c**) in the diagonal of the channel (see left-hand sketches), depending on the fluid velocity.

Figure 4 shows the streamlines and fluid temperature maps, derived from the CFD simulations, for the structure studied and the superficial fluid velocity of 0.3 ms^−1^, 2.0 ms^−1^, and 6.0 ms^−1^, which corresponds to $\mathrm{Re}$ = 89, $\mathrm{Re}\hspace{0.17em}$= 592 and $\mathrm{Re}\hspace{0.17em}$= 1777, respectively. The arrows show the direction of the velocity vectors tangent to the streamline. The black wing-shaped walls of the structure are heated by the electric current thus transferring a heat to the fluid stream. In the CFD approach, the heat flux is constant over the entire surface. Distributions of the velocity vectors and fluid temperatures were considered in three planes for each velocity: a—on the plane along the channel axis; b—on the plane near to the channel walls; c—on the plane in the diagonal of the channel, as shown in Fig. 4.

In the axial plane (Fig. 4a), irrespective of the fluid velocity, the streamlines are parallel and neither inlet nor outlet vortices are formed, moreover, the distribution of the fluid temperatures near the leading edge of the structure indicates intense heat transfer. The heat transfer intensity is proportional to the temperature gradient (see Sect. Methods/CFD simulations at the end of the paper). If the temperature difference between the surface and the core of the flowing fluid is distributed over a very short distance, the temperature gradient is great, hence the intensity of heat transfer is also large; this is the case at the leading edge. On the contrary, near the trailing edge, the temperature gradient is small (the temperature difference between the wall and fluid core is spread over a larger distance), thus less intense heat transfer.

On the plane situated near the channel wall (Fig. 4b) the streamlines indicate the existence of distinct outlet vortices. For the smallest fluid velocity of 0.3 ms^−1^ a vortex covering a relatively large area exists with the centre approximately 1 mm downstream of the channel. For the velocity of 2.0 ms^−1^, the outlet vortex centre is placed about 2 mm downstream of the channel and the vortex is stretched over a wider area. For 6.0 ms^−1^ the vortex is stretched even more; its centre is placed over 3 mm downstream of the channel and within the vortex area, small vortices appear closer to the channel outlet. As judging from Fig. 4b (${w}_{0}\hspace{0.17em}$= 6.0 ms^−1^), the small vortices are unstable as, near the opposite corners, the flow maps differ substantially.

Similar conclusions might be derived from the flow-temperature maps for the diagonal plane (Fig. 4c). For the lowest velocity of 0.3 ms^−1^ the outlet vortex appears as much more intense while for 2.0 ms^−1^, the vortices seem less intense and wider stretched downstream. For the highest velocity of 6.0 ms^−1^, again the small unstable vortices exist. Additionally, an interesting phenomenon is observed in Fig. 4b and c. Within the exit vortex area, the streamlines of the direction opposite to the fluid flow reach the channel wall near the trailing edge and thus enhance the heat transfer. The region of the highest fluid temperature, thus the lowest heat transfer intensity, is shifted toward the channel inlet; this shift increases with the flow velocity almost to the centre of the channel length. As a result, the transport intensity is increased near the trailing edge. Analysis of the outlet vortex streamlines presented in Fig. 4 showed that the outlet vortex range, thus the area of intensified heat transport, expands with increasing fluid velocity from the trailing edge towards the channel inlet by 0.45 mm, 0.78 mm, and 0.97 mm for a fluid velocity of 0.3 ms^−1^, 2.0 ms^−1^ and 6.0 ms^−1^, respectively. This phenomenon of transport intensification by the effect of the outlet vortex is to some extent beneficial for the processes running in monolithic reactors, although the dissipation of energy in this vortex causes some losses. Note, however, that this phenomenon only exists within the corners of the channel, so its impact should be moderate.

**Figure 5:**
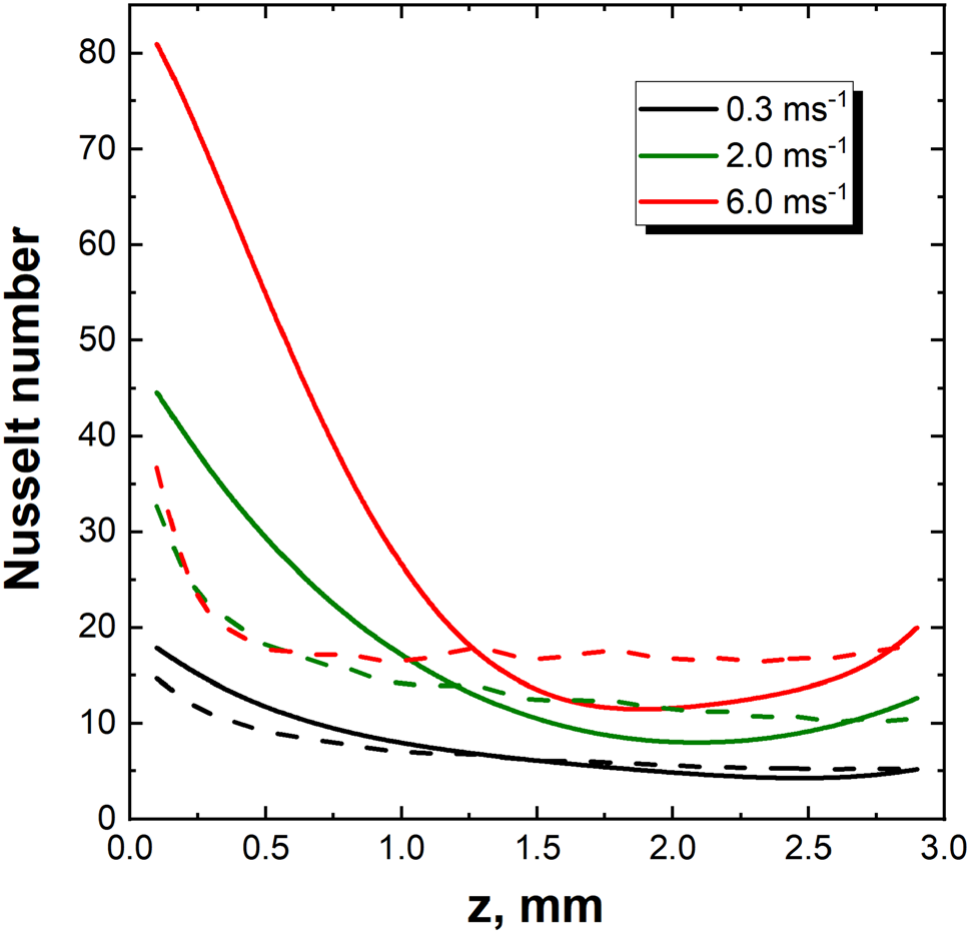
Distribution of the Nusselt numbers (averaged around the circumference) along the channel length). Solid lines—streamlined structure; dashed lines—sharp-edged structure.

The above phenomena are confirmed by the distribution of the local Nusselt number along the channel of the structure (Fig. 5), i.e. the ${\mathrm{Nu}}_{z}$ vs. $z$ dependence, where $z$ is the axis coordinate along the channel and ${\mathrm{Nu}}_{z}$ is averaged around the channel circumference. The local Nusselt number decreases monotonically from the channel inlet towards the outlet and it reaches a certain constant value, then increases near the channel end. This increase is a result of the outlet vortex affecting the boundary layer near the trailing edge of the channel wall as discussed above. The distance of the enhanced heat transfer region (from the trailing edge towards the channel inlet), for a fluid velocity of 0.3 ms^−1^, 2.0 ms^−1^, and 6.0 ms^−1^, amounts to 0.51 mm, 0.85 mm, and 1.12 mm, respectively. This is in satisfactory agreement with the outlet vortex range as discussed above. The distribution of the Nusselt number is close to the theoretical predictions and numerical^4,10^ as well as experimental results^11,12^.

**Figure 6:**
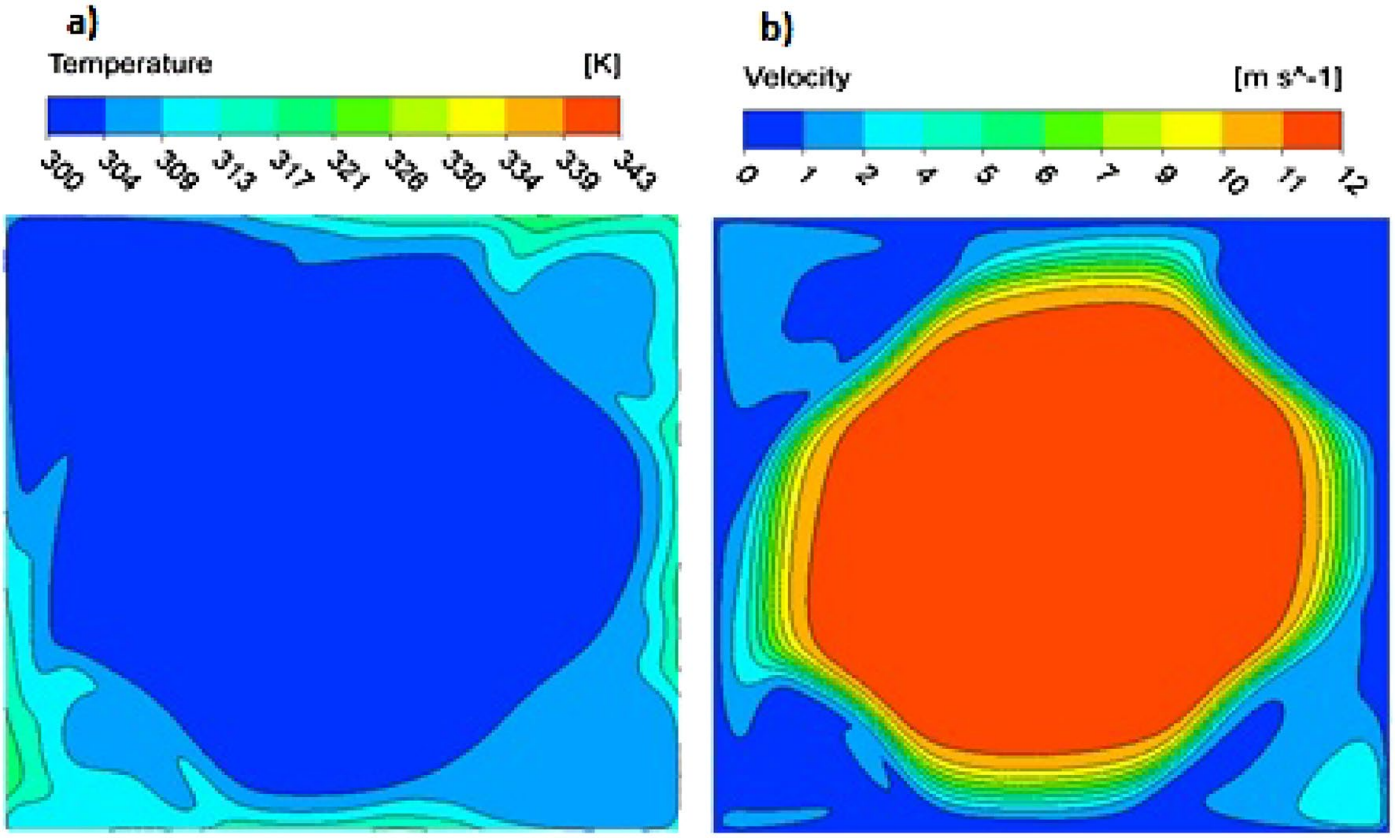
Distribution of the fluid temperature (**a**) and velocity (Fig. 4b) at the channel outlet for streamlined structure, fluid velocity 6.0 ms^−1^ ($\mathrm{Re}\hspace{0.17em}$= 1777).

As visible in Fig. 4, the streamlines for the highest velocities (2.0 ms^−1^ and 6.0 ms^−1^) demonstrate that the region of parallel, laminar flow narrows near the channel walls (see Fig. 4b) while the corners are occupied by the return vortices. Simultaneously major part of the fluid flows in the central part of the channel forming a kind of laminar “core” or “jet”. The atypical distribution of the fluid temperature and velocity at the channel outlet is shown in Fig. 6a and b, respectively, for the highest fluid velocity of 6.0 ms^−1^ ($\mathrm{Re}\hspace{0.17em}$= 1777). The laminar jet area is distinctly visible in Fig. 6b; the velocity attains there 12 ms^−1^, being twice as high as the average velocity in the channel; Reynolds number within the jet exceeds 2500 (considering the jet as a kind of tube). The high velocity gradient around the jet proves intense shear thus rather high pressure drop. In contrast, velocity in the corners is low, within 1–4 ms^−1^. The asymmetric velocity distribution suggests the non-stationary nature of the flow in the corners; this agrees with the results observed in Fig. 4. Temperature distribution near the corners is similar (cf. Fig. 4b,c); it shows rather weak intensity of heat transfer.

**Figure 7:**
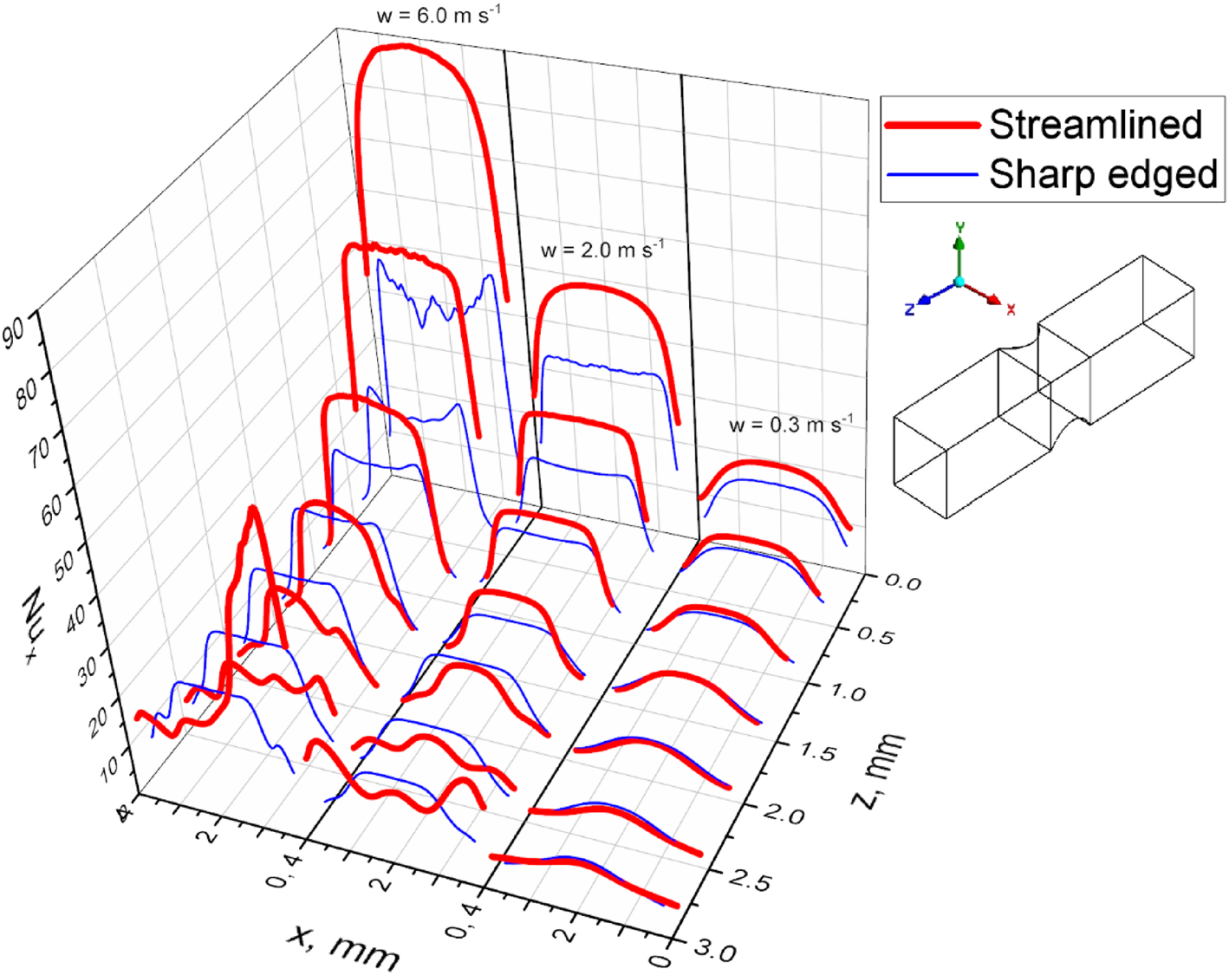
Distribution of the local Nusselt number ${\mathrm{Nu}}_{x}$ along the channel wall for the streamlined and sharp-edged structures.

These phenomena affect the distribution of transport intensity, i.e. the local Nusselt number, along the channel wall (${\mathrm{Nu}}_{x}$ dependence on $x$, where $x$ is the transverse coordinate in the channel) as presented in Fig. 7.

For the lowest velocity of 0.3 ms^−1^ ($\mathrm{Re}\hspace{0.17em}$= 89), the transverse ${\mathrm{Nu}}_{x}$ distribution is similar to the theoretical one for the developing laminar flow; the Nusselt number distribution is almost flat within the channel centre and decreases towards the corners (more rapidly near the inlet, moderately near the outlet). The transport intensity decreases with the distance $z$. CFD study^10^ shows temperature distribution for developing flow in square channels (assuming no wall impact) suggesting similar ${\mathrm{Nu}}_{x}$ distribution.

For 2.0 ms^−1^ ($\mathrm{Re}$ = 592), approximately up to $z\hspace{0.17em}$= 1.5mm the ${\mathrm{Nu}}_{x}$ vs. $x$ dependence is similar. Deeper in the channel, say for $z$ > 1.5mm, the relationship flattens out near the corners, and for $z$ > 2 mm local maxima appear there, right at the outlet even higher than the ${\mathrm{Nu}}_{x}$ value in the centre. This is a result of the above-mentioned outlet vortex influence on the channel walls near the corners (cf. Fig. 4a,c).

For the highest velocity of 6.0 ms^−1^ ($\mathrm{Re}$ = 1777), the distribution of the Nusselt number becomes distorted near the corners already from $z$ = 1.5 mm. The distribution becomes asymmetric and local maxima appear near the corners. Near the channel outlet, the distribution is strongly asymmetric, which proves that the phenomena are non-stationary. The exceptionally high maximum is temporary; in the opposite corner, the value of the Nusselt number is much lower. Although the Reynolds number equal to 1777 suggests laminar flow in the channels of the structure, vortices at the outlet indicate significant inertial effects (cf. Fig. 4). Also the laminar jet at the channel outlet (cf. Fig. 6) reaching a velocity of over 12 ms^−1^ and Reynolds number over 2500 within the jet core proves strong inertial interactions. The result is the unusual transverse distribution of transport intensity shown in Fig. 7, which is more characteristic of the turbulent rather than laminar flow.

### Sharp-edged structure

**Figure 8:**
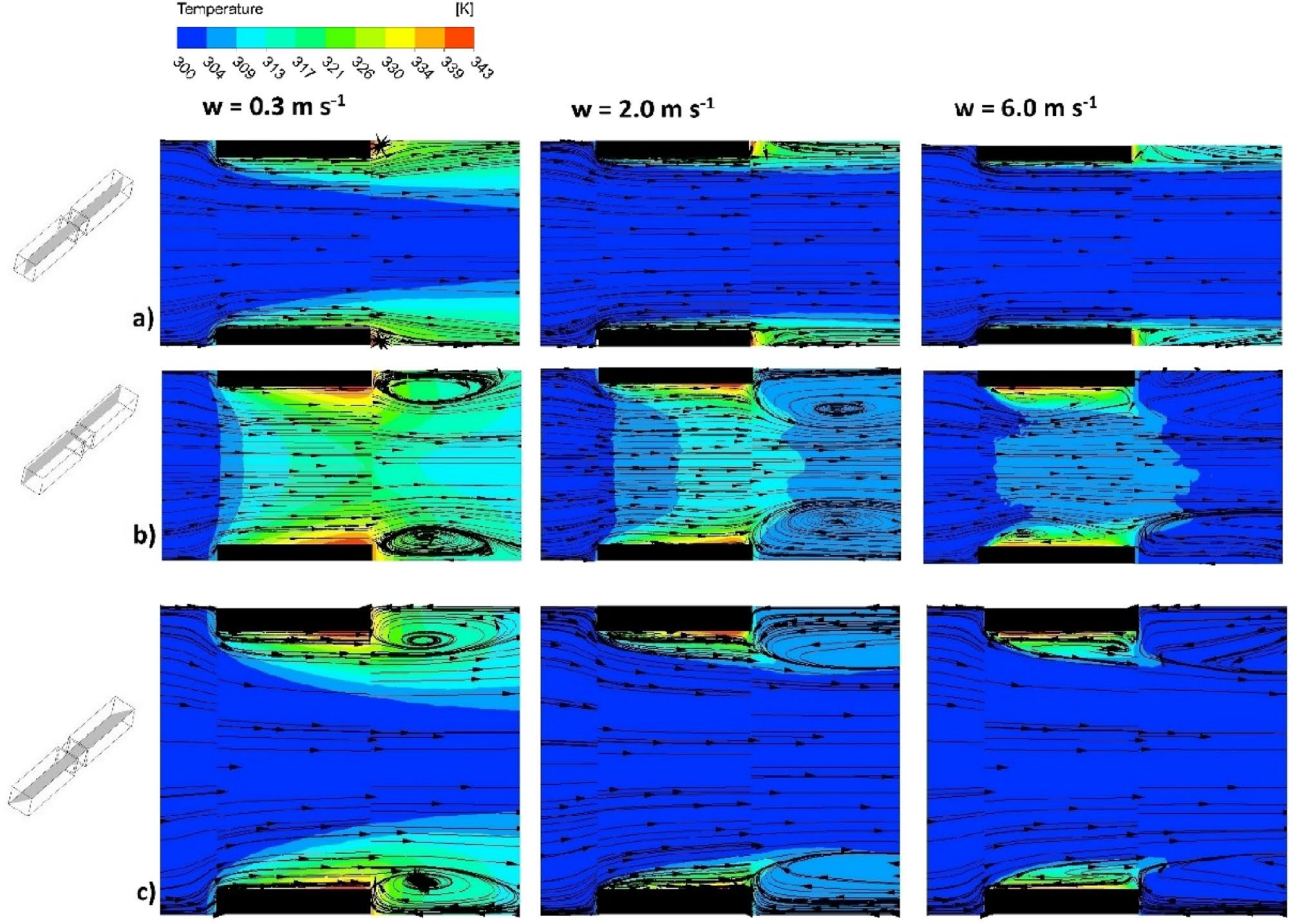
Streamlines and fluid temperature maps for the sharp-edged structure: (**a**) on the plane along the channel axis; (**b**) on the plane near to the channel wall; (**c**) on the plane in the diagonal of the channel (cf. left-hand sketches).

To compare the phenomena of flow and transfer, the same CFD simulation program as for streamlined structure was performed for the sharp-edged one. The streamlines and the fluid temperature maps are presented in Fig. 8. The simulations were performed for the same gas velocities as for the streamlined structure (cf. Fig. 4). In Fig. 8, the superficial fluid velocity of 0.3 ms^−1^, 2.0 ms^−1^ and 6.0 ms^−1^ corresponds to $\mathrm{Re}\hspace{0.17em}$= 79, $\mathrm{Re}$ = 529, and $\mathrm{Re}\hspace{0.17em}$= 1587, respectively. Moreover, the longitudinal and transverse distributions of the Nusselt number for the sharp-edged structure are presented in Figs. 5 and 7, respectively.

As judging from Fig. 8, in all the planes considered intense outlet vortices are formed starting from the lowest velocity (0.3 ms^−1^). The inlet vortex is distinct for the velocity of 2.0 ms^−1^ especially in the corners (Fig. 8b,c), but also in the middle of the wall (Fig. 8a). For 6.0 ms^−1^ the inlet vortices cover the entire length of the channel in the area of the corners and half the length in the middle part of the wall; some asymmetries of the streamlines, thus flow instabilities, appear then both within the inlet and outlet vortices.

The inlet vortices to some extent shield the channel walls near the inlet, which may result in local minima of the Nusselt number distribution in Fig. 5. The minima are not distinct there; they might appear at a distance of approx. 0.7–1.2 mm from the channel inlet where the Nusselt distribution is slightly “wavy”. The existence of the local minima of the Nusselt number is confirmed by the higher wall temperature within the inlet zone, even higher than near the outlet (Fig. 8b, 6.0 ms^−1^). For the sharp-edged structure, the increase in the Nusselt number near the outlet is less pronounced than for streamlined ones (cf. Fig. 5).

The transverse distribution of Nusselt number (${\mathrm{Nu}}_{x}$ vs. $x$) for the sharp-edged structure and the velocity of 0.3 ms^−1^ is close to that of the streamlined ones (cf. Fig. 7). For higher velocity, 2.0 ms^−1^, near the inlet, weak instabilities appear on the transverse Nusselt distribution as well as slight minima in the middle of the channel wall; no local maxima within the corners exist. Near the outlet, the distribution is close to the theoretical predictions^4^.

For the highest velocity studied, 6.0 ms^−1^, strong instabilities at the transverse Nusselt distribution exist near the channel inlet and distinct local minima appear there (Fig. 7). This is the effect of a strong inlet vortex covering a large part of the channel near the inlet, especially within the corners (cf. Fig. 8b,c, $w$ = 6.0 ms^−1^). Further down the channel, the distribution becomes more regular and, near the outlet, slight local minima and maxima appear in the corners, an effect of the intense outlet vortex (Fig. 8).

### Streamlined vs. sharp-edged structures: a comparison

While comparing Figs. 4 and 8, the fundamental differences between streamlined and sharp-edged structures are visible. For the streamlined structures, there is no inlet vortex (Fig. 4) regardless of the flow velocity, while such a vortex appears for the sharp-edged structures near the channel inlet for higher velocities (Fig. 8).

Outlet vortices occur for both structures, most intense in the channel corners. For the streamlined structure, the outlet vortex affects the channel wall near the outlet thus intensifying heat transport in this area, as evidenced by the streamlines in Fig. 4 and the longitudinal distribution of the Nusselt number (Fig. 5). Such a phenomenon for sharp-edged structure is barely noticeable (Fig. 8).

The transverse distributions of the Nusselt number for both structures differ significantly (Fig. 7). For the streamlined structure, the distribution in the inlet channel section is close to theoretical predictions, a result of the lack of the inlet vortex. In the outlet part, however, as a result of the outlet vortex impact, distinct local maxima of the Nusselt number appear, mainly in the corners. For the sharp-edged structure, for higher flow velocities, the most distinct irregularities appear near the channel inlet. Local minima and maxima and their irregular course prove the non-stationarity of the flow in this region as the result of strong interactions of the inlet vortex. On the other hand, in the outlet area, the distribution of the Nusselt number is close to the theory; only for 6.0 ms^−1^, small local maxima appear in the corners near the outlet. To sum up, the streamlined structure is close to the theory at the channel inlet while deviations occur at the outlet; on the contrary, the sharp-edged structure reflects the theory at the outlet, and the most serious deviations appear near the inlet.

**Figure 9:**
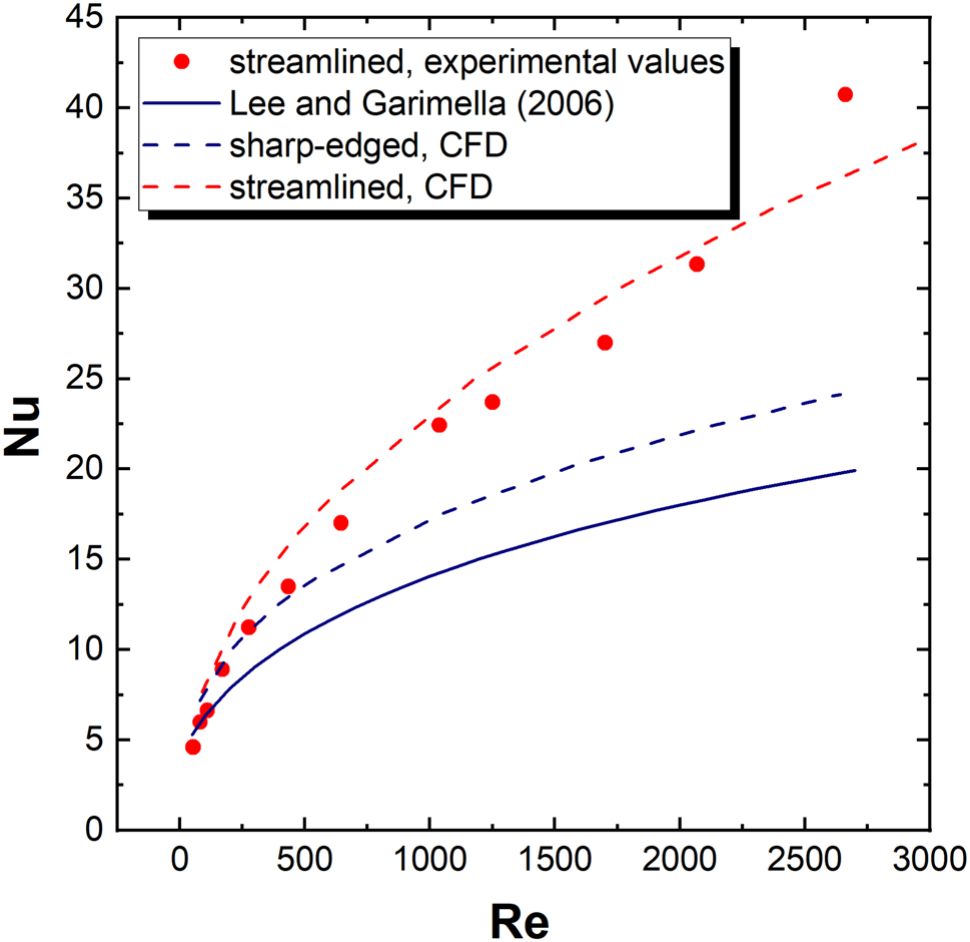
Experimental, CFD derived, and literature correlation (Lee and Garimella^13^) of Nusselt number (averaged) vs. Reynolds number for streamlined and sharp-edged structures.

Figure 9 shows the dependences of the Nusselt number (averaged over the whole channel surface) vs. the Reynolds number. The experimental and CFD results for the streamlined structure are compared with the CFD data for the sharp-edged structure. Additionally, the literature correlation of Lee and Garimella^13^ is presented. Figure 9 proves that the heat transfer for streamlined structures is more intense compared with the sharp-edged ones.

## Conclusions

Experimental tests and CFD simulations for the developed streamlined structure with a square cross-section and CFD tests for the classic structure (sharp-edged) with a similar geometry were carried out. Experimental results of heat transfer studies showed satisfactory agreement with CFD simulations.

For the streamlined structure, there is no inlet vortex, while the distribution of the Nusselt number near the inlet of the channel is close to the theoretical relationships. For the sharp-edged ones, intense inlet vortices exist that screen the channel wall and cause a local Nusselt number minima in the inlet channel zone (Fig. 5). There, distinct and irregular maxima and minima appear at the transverse Nusselt distribution (Fig. 7) which testify to the non-stationarity of the flow in this zone.

Intense outlet vortices exist for both types of structures. For the streamlined structure, higher fluid velocities, affect the channel wall near the outlet, thus intensifying heat transport in this area, as evidenced by the local maxima of the Nusselt number in Figs. 5 and 7. For the sharp-edged structure, such interactions are much weaker.

The main difference of the flow patterns between both types of structures is the interaction of the inlet and outlet vortices. For the streamlined structure, the inlet vortex does not exist, while the outlet vortex affects (for higher velocities) the channel walls in the outlet zone. As a result, for the streamlined structure, the distribution of streamlines and Nusselt numbers is close to the theory in the inlet zone, while deviations appear near the outlet (local maxima and minima of the Nusselt number, see Figs. 5 and 7).

For the sharp-edged structure, the strong inlet vortex causes significant disturbances of the streamlines, and the Nusselt number distributions show significant deviations from the theory, like local minima and irregular waveforms. On the other hand, the flow in the outlet zone is close to theoretical predictions, because the impact of the otherwise strong outlet vortex on the surface of the channel is much weaker (cf. Figs. 5 and 7). As the final remark, the heat transfer intensity is better for the streamlined structure (as judging from Fig. 5).

## Methods

### Experimental set-up

The experimental program of heat transfer was realized. All experiments were performed using air in ambient conditions and superficial fluid velocity wo within 0.3–6.0 ms^−1^ (Reynolds number 89–1777). The experimental reactor had a rectangular cross-section, 30 × 45 mm. The metal structure was heated using a strong electric current flowing directly through it (exploring the Joule effect); this has led to approximately constant heat flux on the surface of the structure, corresponding to the H1 boundary condition (constant axial wall heat flux with constant peripheral wall temperature^4^). The air stream temperature was measured by thermocouples (three placed before the structure and three behind it). The temperature of the surface of the structure was measured by eight thermocouples (four at the inlet side of the structure and four at the outlet one), and the accuracy was 0.2 K. The thermocouples were attached to the structure with epoxy glue, providing good thermal conductivity and complete electrical insulation. A more detailed description of the experimental procedure and the scheme of the experimental set-up are presented in former paper^14^.

The heat transfer coefficient, $h$, was calculated according to the equation:

1 $$h=\frac{Q}{F\Delta {T}_{m}}$$

where $Q$—heat exchanged (W); $F$—heat exchange surface (m^2^); $\Delta {T}_{m}$—logarithmic mean of inlet and outlet temperature differences between the flowing fluid and the structure surface.

Reynolds number, $\mathrm{Re}$, was defined as:

2 $$\mathrm{Re}=\frac{w\rho {D}_{h}}{\mu }$$

where $w=({w}_{0} {\varepsilon }^{-1})$ is interstitial fluid velocity, (ms^−1^); $\rho$ is fluid density, (kg m^−3^); $\mu$ is fluid viscosity, (kg m^−1^s^−1^).

Nusselt number Nu was defined as:

3 $$\mathrm{Nu}=\frac{h{D}_{h}}{k}$$

where $k$ is thermal conductivity, (W m^−1^ K^−1^).

Although mass transfer has not been investigated either experimentally or numerically (CFD) in the presented research, note that it is directly related to heat transport by the Chilton–Colburn analogy:

4 $$\frac{\mathrm{Sh}}{\mathrm{Nu}}=\frac{{\mathrm{Sc}}^\frac{1}{3}}{{\mathrm{Pr}}^\frac{1}{3}}$$

where $\mathrm{Sh}={k}_{C}{D}_{h}{D}_{A}^{-1}$ is Sherwood number, *k*_*C*_—mass transfer coefficient (ms^−1^),—${D}_{A}$ diffusivity (m^2^s^−1^), $\mathrm{Sc}$—Schmidt number, and $\mathrm{Pr}$—Prandtl number.

### CFD simulations

The numerical CFD simulations of the fluid flow and heat transfer were carried out using the Ansys Fluent software. The model concerned a single 3 mm long channel. The polyhedral grid for the streamlined structure consisted of 2.46 million elements while the sharp-edged one of 467,000 elements. The mesh was generated in the Ansys meshing program. The steady-state calculations were performed using a pressure-based solver and laminar flow model. The coupled scheme method was used for pressure–velocity coupling, and the Green–Gauss Cell-Based method for gradients evaluation. Second-order upwind discretization schemes were applied for pressure, momentum, and energy. Air was chosen as a model fluid with temperature-independent physical properties. The velocity and temperature profiles were uniform at the channel inlet. Constant heat flux was defined on the channel walls. Convergence was assured by monitoring the scaled residuals at a constant level below 10^–3^ for each variable, except the energy residual, for which the criterion was 10^–6^. Post processing was performed at Ansys CFD-Post. More details are available in^14^.

As the constant heat flux $\left(\dot{q}=constant\right)$ was assumed, the local heat transfer intensity was characterized by the local heat transfer coefficient, $h$, and the local temperature gradient, $\mathrm{grad }T={\left.\frac{dT}{dy}\right|}_{y=0}$ where $y$ is the coordinate perpendicular to the channel wall (heat transfer surface):

5 $$\dot{q}=h\Delta T=-k{\left.\frac{dT}{dy}\right|}_{y=0}=constant$$

where $\Delta T$ is the temperature difference between surface and bulk fluid. Thus the local heat transfer coefficient, $h$, is proportional to the local temperature gradient, $h=-\frac{k}{\Delta T}{\left.\frac{dT}{dy}\right|}_{y=0}$.

## Data Availability

All data generated during this study (both experimental and CFD) have been presented in graphical form in the paper.
